# Lipid droplets in plants: turnover and stress responses

**DOI:** 10.3389/fpls.2025.1625830

**Published:** 2025-06-27

**Authors:** Yujie Zhao, Rui Cao, Jincheng Li, Yingying Xu, Lijuan Zhou, Yajin Ye

**Affiliations:** State Key Laboratory of Tree Genetics and Breeding, National Key Laboratory for the Development and Utilization of Forest Food Resources, Co-Innovation Center for Sustainable Forestry in Southern China, Nanjing Forestry University, Nanjing, China

**Keywords:** lipid droplets, biogenesis, degradation, stress responses, SDP1, lipophagy

## Abstract

Lipid droplets (LDs) have emerged as dynamic organelles central to plant lipid metabolism, stress adaptation, and energy homeostasis. This review synthesizes recent advances in understanding LD biogenesis and degradation in plants, highlighting conserved and divergent mechanisms relative to other eukaryotes. LD formation originates in the endoplasmic reticulum (ER), where neutral lipids synthesized by diacylglycerol acyltransferases (DGAT) and phospholipid: diacylglycerol acyltransferases (PDAT) accumulate into lens-like structures. These structures bud into the cytosol via ER machinery, including SEIPIN complexes, vesicle-associated membrane proteins, and LD-associated protein-interacting protein which regulate LD size and abundance. Degradation occurs through two major pathways: lipolysis, mainly mediated by the patatin-like lipase SUGAR-DEPENDENT1, and lipophagy, where AUTOPHAGY-RELATED proteins deliver LDs for breakdown. LDs also function as stress-responsive hubs, accumulating under abiotic stresses and during pathogen interactions, where they participate in membrane remodeling and antimicrobial defense. Extensive studies in major oilseed crops reveal that expressions of multiple genes involved in LD turnover are significantly induced under various abiotic stresses and phytohormone treatments. These genetic components operate autonomously or synergistically (e.g. DGAT and PDAT) within the TAG biosynthesis and LD metabolic pathways, effecting concurrent enhancements in stress resilience and oil production under suboptimal growth conditions. Critical knowledge gaps persist, including the interplay between lipolysis and lipophagy, the integration of energy-related signaling pathways in LD turnover, and stress-modulated post-translational control of LD proteome. Deciphering these mechanisms will advance our understanding towards LD biology.

## 1 Introduction

Since their identification as organelles in the 19th century, lipid droplets (LDs) have undergone various nomenclature changes. They were once referred to as lipid bodies, adiposomes, oil bodies, sphaerosomes and oleosomes, but are now commonly known as LDs (Walther and Farese, 2012). LDs are lipid-rich organelles, which possess a core of neutral lipids, predominantly triacylglycerols (TAGs) and steryl/wax esters, which is encased by a monolayer of phospholipids (PLs).

LDs are derived from the endoplasmic reticulum (ER), and the biogenesis of LDs includes the following key steps: neutral lipid synthesis at the ER; formation of a lipid lens; budding of LDs; LD growth and maturation (Mathiowetz and Olzmann, 2024). In the last decade, proteins involved in these steps have been well characterized in plants, especially model plant *Arabidopsis thaliana*. These proteins include TAG-synthesizing enzymes and the proteins responsible for the LDs generation, such as SEIPIN, VESICLE-ASSOCIATED MEMBRANE PROTEIN-ASSOCIATED PROTEIN 27 (VAP27) and LD-ASSOCIATED PROTEIN-INTERACTING PROTEIN (LDIP) (Barneda and Christian, 2017; Man et al., 2024). The degradation of LDs in plants is also a tightly regulated process, mainly mediated by lipolysis and lipophagy. Among the key players in lipolysis, a conserved patatin domain containing protein SUGAR-DEPENDENT1 (SDP1) stands out (Eastmond, 2006; Huang et al., 2022). In *A. thaliana* leaves, lipophagy occurs through microautophagy, relying on the core components of the macroautophagy pathway (Fan et al., 2019a).

The role of LDs in carbon reserve storage is fundamental to the survival and growth of plants. However, over the past decade, a paradigm shift has occurred in the perception of LDs in plant biology. Except acting as static storage organelles, LDs are now recognized as dynamic subcellular structures actively involved in multiple physiological processes. Mounting evidence has shown that LDs play a crucial role in stress adaptation. Under abiotic stress conditions such as drought, cold, and heat stress, the abundance of LDs increases in plant cells (Yang et al., 2011; Kong et al., 2013; Kim et al., 2016; Yang et al., 2024). This new understanding has highlighted the importance of lipid metabolism, lipid transport, and stress responses in plants.

Given the significance of LDs in plant physiology, this review aims to provide a comprehensive overview of the latest research advancements in the biogenesis and degradation of LDs in plants. It will also explore the importance of LDs in the stress response of plants. By integrating findings from recent studies, we hope to shed light on the complex molecular and physiological processes associated with LDs in plants, which may have implications for crop improvement, bioenergy production, and understanding plant responses to environmental changes.

## 2 The proteins involved in the generation of lipid droplets

LD biogenesis in plant cells shares conserved mechanisms with other eukaryotes, relying on ER-localized protein machinery to initiate LD formation and on LD surface proteins to ensure proper cytoplasmic packaging. The process begins with the synthesis of neutral lipids within the ER, where they accumulate into lens-like structures between the ER membrane leaflets (Scholz et al., 2022). Key proteins, such as SEIPIN and VAP27, facilitate the budding of nascent LDs into the cytoplasm (Guzha et al., 2023). During this step, the phospholipid monolayer of the LD becomes continuous with the outer ER membrane leaflet. Subsequently, additional proteins, including lipins and LD coat proteins, are recruited to promote LD growth. However, the mechanism underlying LD dissociation from the ER remains poorly understood (Bouchnak et al., 2023).

### 2.1 Enzymes for neutral lipids synthesis

**Figure 1:**
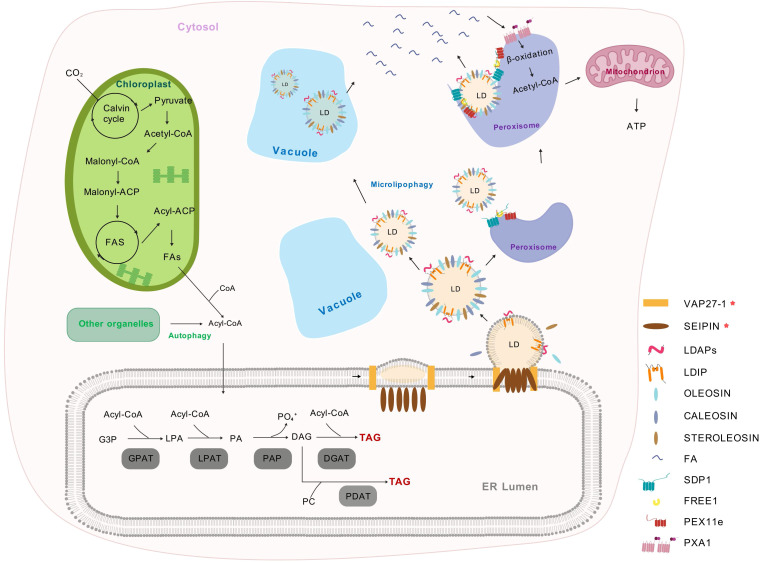
The biosynthesis and metabolism of plant lipid droplets (Park et al., 2013; Choi et al., 2022; Huang et al., 2022; Guzha et al., 2023). Plastids supply FAs that are transported to the cytosol and activated into Acyl-CoA. In the endoplasmic reticulum, G3P is acylated to form LPA using Acyl-CoA. LPA is further acylated by LPAT to produce PA. PAP dephosphorylates PA to DAG, which can be acylated by DGAT to form TAG. DAG can also exchange with PC, which are generated through an acyl editing cycle involving reacylation and acylation. LDs store TAG and are covered by a single layer of phospholipids and LD-associated proteins. The budding of LDs from the ER is regulated by the SEIPIN protein complex (including SEIPIN1, SEIPIN2, and SEIPIN3), which acts as an ER-localized scaffold protein to ensure proper LD formation by controlling neutral lipid synthesis and droplet size. Additionally, VAP27–1 functions as an ER-LD contact site protein, mediating phospholipid transfer to promote LD maturation and stabilize the LD formation complex. As LDs mature, they recruit proteins such as LDAP and LDIP, OLEOSIN, CALEOSIN, and STEROLEOSIN, which contribute to LD structure, stability, and function. During lipolysis, the ESCRT component FREE1 directly interacts with both PEX11e and SDP1, thereby regulating SDP1-mediated LD degradation and promoting FAs release. And these FAs are transported into peroxisome by PXA1 for *β*-oxidation. In contrast, lipophagy involves the selective autophagy of LDs, delivering them to vacuoles for breakdown. FAs, fatty acids; G3P, glycerol-3-phosphate; LPA, lysophosphatidic acid; LPAT, lysophosphatidic acid acyltransferase; PA, phosphatidic acid; PAP, Phosphatidic acid phosphatase; DAG, diacylglycerol; DGAT, acylated by diacylglycerol acyltransferase; PC, phosphatidylcholines; LDs, lipid droplets; TAG, triacylglycerol; ER, endoplasmic reticulum; VAP27-1, VESICLE-ASSOCIATED MEMBRANE PROTEIN-ASSOCIATED PROTEIN 27-1; LDAP, LIPID DROPLET-ASSOCIATED PROTEIN; LDIP, LDAP-INTERACTING PROTEIN; ESCRT, ENDOSOMAL SORTING COMPLEX REQUIRED FOR TRANSPORT; FREE1, FYVE DOMAIN PROTEIN REQUIRED FOR ENDOSOMAL SORTING 1; PEX11e, PEROXIN 11e; SDP1, SUGAR DEPENDENT 1; PXA1, PEROXISOMAL ABC TRANSPORTER 1.

In plants, the ER serves as the principal site for TAG biosynthesis, which is mainly accomplished through the glycerol-3-phosphate (G3P) pathway or the Kennedy pathway (Xu and Shanklin, 2016). Firstly, glycerol-3-phosphate acyltransferase (GPAT) catalyzes the combination of G3P and Acyl-CoA, resulting in the formation of lysophosphatidic acid (LPA). Subsequently, under the catalytic action of lysophosphatidic acid phosphatase (LDPAT), LPA combines with Acyl-CoA once more to produce phosphatidic acid (PA). Phosphatidic acid phosphatase (PAP) then dephosphorylates PA to generate diacylglycerol (DAG). Finally, DAG undergoes final acylation to form TAG through two distinct mechanisms. The Acyl-CoA-dependent pathway, catalyzed by diacylglycerol acyltransferases (DGATs), utilizes Acyl-CoA as the acyl donor (Walther and Farese, 2012). Alternatively, phospholipid: diacylglycerol acyltransferase (PDAT) drives an Acyl-CoA-independent route by transferring an acyl moiety from phosphatidylcholines (PC) to DAG, producing TAG alongside a lysophospholipid (Bates et al., 2013). Thereafter, TAGs are subsequently stored between the two leaflets of the ER. As TAG accumulates and LDs enlarge, they separate from the ER membrane and enter the cytoplasm (**Figure 1**, **Table 1**).

**Table 1:** Functionally characterized genes involved in LD turnover in plants.

Pathway	Gene types(molecular function)	Species	Gene names	Growth and development related functions	Stress-response-related functions	References
TAG Synthesis	GPATs (catalyze the combination of G3P and Acyl-CoA, resulting in the formation of LPA)	*A. thaliana*	*AtGPAT4/8*	Involved in cuticle development through regulating lipid homeostasis	Mediate plant immune responses through pathogen-induced dynamic relocation of LDs	Fernández-Santos et al., 2020
	DGATs (catalyze DAG acylation with Acyl- CoA to synthesize TAG)	*A. thaliana*	*AtDGAT1/2*	*dgat1-1* and *dgat1-1 dgat2* lines exhibit reduced seed oil content	Stabilize plasma membrane integrity and enhance thermotolerance	Zhang et al., 2009; Regmi et al., 2020, Shomo et al., 2024;
		*G. max*	*GmDGAT1-2*	Significantly increases seed oil content and oleic acid (18:1) accumulation during TAG biosynthesis		Jing et al., 2021; Torabi et al., 2021; Xu et al., 2021
		*Jatropha curcas*	*JcDGAT1/2*	Enhance seed oil accumulation while reducing protein and soluble sugarcontent		Zhang et al., 2021
		*Plukenetia volubilis* L.	*PvDGAT2-2*	Catalyzes TAG biosynthesis in leaves	Confers thermotolerance through MYB1-dependent lipid remodeling in plants	Yang et al., 2024
	PDATs (transfer an acyl moiety from PC to DAG, producing TAG)	*A. thaliana*	*AtPDAT1*	Delays plant senescence by regulating fatty acid turnover, membrane lipid homeostasis and TAG synthesis	Enhances tolerance to temperature stress	Fan et al., 2019b; Demski et al., 2020; Shomo et al., 2024
LD Synthesis	VAP27 (interacts with the N terminus of SEIPIN2 and/or SEIPIN3 to stabilize the LD- forming complex)	*A. thaliana*	*AtVAP27-1*	*vap27-1* mutants reveal a large LD phenotype in seeds	Enhances ER stress resistance by modulating ER-PM contact sites and calcium homeostasis	Siao et al., 2016; Greer et al., 2020; Man et al., 2024
		*Z. mays*	*ZmVAP27-1*	Supports cellular expansion and normal growth processes by regulating aquaporin activity	Maintains water homeostasis and minimizes ionic imbalance, enhancing salinity tolerance	Fox et al., 2020
		*Populus tremula ×tremuloides*	*PttVAP27-17*	Improves carbon allocation and energy storage; Promotes plant growth and development	Mediates stress-responsive energy mobilization to sustain plant survival	Gandla et al., 2021
	SEIPINs (interact with LDIP to modulate the number and size of LDs, facilitating LD biogenesis)	*A. thaliana*	*AtSEIPIN1/2/3*	Expression of *SEIPIN1* promotes accumulation of large-sized LDs, while expression of *SEIPIN2* and *SEIPIN3* promote small LDs		Cai et al., 2015
		*Thlaspiarvense*	*TaSEIPIN1/2/3*	Involved in the YELLOW and MATURE late seed maturation stages		Luján et al., 2025
	LDAPs (LD coat protein, regulate LD compartmentation)	*A. thaliana*	*AtLDAP1/2/3*	Formation and expansion of LDs in leaves	LDAP1 regulate LD dynamics in response to heat stress; LDAP3 is involved in cold tolerance	Gidda et al., 2016
		*Z. mays*	*ZmLDAP1/2*	Regulates LD clustering	Participates in plant antiviral defense by regulating C18 polyunsaturated fatty acid (PUFA) biosynthesis	Wang et al., 2024
		*Populus trichocarpa*	*PtLDAP1/2/3*	Promote lipid body expansion through binding to the expanding monolayer		Veerabagu et al., 2021
	LDIP (interacts with LDAPs and SEIPINs topromote LD formation)	*A. thaliana*	*AtLDIP*	Regulates LD compartmentation inseeds, seedlings and leaves		Pyc et al., 2017
		*T. arvense*	*TaLDIP*	*Taldip* mutants exhibite increased seed oil content without compromising plant growth		Guzha et al., 2024
	LDPS (Interacts with OLE1 to regulate fusion)	*A. thaliana*	*AtLDPS*	Promotes LD expansion and maintains seed oil content		Doner et al., 2025
	OLEOSINs (regulate LD size)	*A. thaliana*	*AtOLE1*	Maintains LD size homeostasis in pollen and seeds	Adaptive modification of LDs for freezing tolerance	Siloto et al., 2006; Miquel et al., 2014; Doner et al., 2025
		*P.trichocarpa*	*PtOLE6*	Involved in LD production, enlargement		Veerabagu et al.,2021
		*G. max*	*GmOLE1*	Stabilizes oil body structure to inhibit lipid release; Enhances seed development		Cao et al., 2015; Jing et al., 2021
		*Caryacathayensis*	*CcOLE2*	Mediates embryonic maturation processes		Chen et al., 2023
	CALEOSIN (mediates overlapping functions in oil accumulation)	*A. thaliana*	*AtCLO1/2*	Mediate lipophagy to regulate LD catabolism and acyl remodeling in germinating seeds	Facilitates antimicrobial compound production to enhance biotic stress resistance	Chen et al., 1999; Wang et al., 2017; Miklaszewska et al., 2023
		*G. max*	*GmCLO1*	Potentially affects soybean reproductive development	Knockout *GmCLO1* elevates pest resistance, where as over expression lines compromisesdefense	Cai et al., 2025
		*O. sativa*	*OsCLO5*	RNAi lines of *OsClo5* have higher survival than WT seedlings	Regulates plant cold resistance through inhibition of JAsignalling and synthesis	Zeng et al., 2022
	Steroleosin/HSD (is involved in the brassinosteroids-related pathway)	*A. thaliana*	*AtHSD1*	Involved in BRs biosynthesis and degradation in seeds and seedlings	Regulates stress responses via hormone signaling	Shao et al., 2019
		*C.cathayensis*	*CcHSD5*	Promotes embryonic development		Chen et al., 2023
		*Pinus massoniana*	*PmHSD-A/B*	Stabilize oil body structures via its sterol-binding domain and exhibits sterol-coupled dehydrogenase activity		Pasaribu et al., 2016
		*O. sativa*	*OsHSD1*	Modulates wax metabolism; Regulates plant height and leaf cuticle development	Is induced by salt and cold stress, potentially mediating abiotic stress responses	Shao et al., 2019
LD Degradation	SDP1 (catalyzes the hydrolysis of TAG inLDs)	*A. thaliana*	*AtSDP1*	*sdp1* mutants exhibit apostgerminative growth arrestphenotype, which can be rescued by providing sugar	Disruption of *SDP1* enhances plant tolerance to darkness	Eastmond, 2006; Fan et al., 2017
		*G. max*	*GmSDP1*	Negatively regulates seed oil content and fatty acid composition	Enhances drought resistance by regulating stress-induced TAG hydrolysis in cotyledons	Kanai et al., 2019; Aznar-Moreno et al., 2022; Xing et al., 2023
		*Brassicanapus* L.	*BnSDP1*	RNAi lines show enhanced seed oilyield without compromising vigor		Kelly et al., 2013b
		*J. curcas*	*JcSDP1*	Gene silencing of *JcSDP1* enhances seed oil accumulation in seeds		Kim et al., 2014
	PUX10 (collaborates with CDC48 to facilitate the degradation of ubiquitinated proteins)	*A. thaliana*	*AtPUX10*	Drives seed germination	Participates in stress-responsive transient metabolic regulation via phase-separated condensate formation	Deruyffelaere et al., 2018; Liu et al., 2024
		*C.cathayensis*	*CcPUX10*	Promotes seed development	Modulates LD biogenesis and stability	Chen et al., 2023
	OBL1 (a TAG lipase associated with LDs)	*A. thaliana*	*AtOBL1*	Facilitates rapid pollen tube growth	Maintains cellular homeostasis by preventing free FAs toxicity	Müller and Ischebeck, 2018
		*Nicotiana benthamiana*	*NtOBL1*	Hydrolyzes LD-stored TAGs to supply membrane lipids; Mediates pollen tube growth	Bypasses *β*-oxidation by directly channeling FAs to the ER for hypoxic adaptation	Müller and Ischebeck, 2018
		*Avena sativa* L	*AsOBL1-like*	Affects vegetative morphogenesis	Orchestrates stress-responsive lipid metabolism for flag leaf adaptation	Li et al., 2025
	ATG (regulates autophagosome biogenesis and autophagy)	*A. thaliana*	*AtATG5/7*	Promotes nitrogen remobilization; Boosts autophagy-driven nutrient recycling	Delays leaf senescence, maintains adaptability under nutrient and drought stresses	Xia et al., 2012; Marshall and Vierstra, 2018
		*N.benthamian a*	*NbATG5/7/8*	Maintains normal development and suppresses leaf malformation	Degrades viral silencing suppressors, boosting antiviral defense	Haxim et al., 2017; Marshall and Vierstra, 2018
		*Populusalba × Populus glandulosa*	*PagATG18*	Promotes xylem lignification	*PagATG18* overexpress linesenhance salt tolerance and reduces oxidative membrane	Courtois-Moreau et al., 2009; Yu et al., 2023
		*O. sativa*	*OsATG5/7*	Deficiency of *ATG5/7* and related genes compromise pollen fertility, impairing reproductive development	*ATG8* overexpress lines enhance drought tolerance via autophagy-mediated resource recycling	Kurusu et al., 2014; Marshall and Vierstra, 2018
		*Z. mays*	*ZmATG6/8a/10*	Facilitates remobilization of N/K/Zn to leaves	Promotes salt tolerance through enhanced autophagy	Li et al., 2015; Khan et al., 2024
		*Triticum aestivum*	*TaATG8*	Autophagy deficiency induces premature floret abortion, severely impairing reproductive growth	Enhances stress resilience by coordinating autophagy, iron homeostasis, and defense signaling	Ghiglione et al., 2008; Yue et al., 2022
	PXA1 (transports FAs into peroxisomes for *β*-oxidation)	*A. thaliana*	*AtPXA1*	*pxa1* mutant fails to germinate onsucrose-free medium; Extended dark conditions are lethal for *pxa1* plants	*pxa1-2* and *pxa1-3* mutants exhibit enhanced salt tolerance due to reduced ROS accumulation	Park et al., 2013; Fan et al., 2017; Yu et al., 2019
	CDC48 (facilitates the unfolding and removal of membrane proteins)	*A. thaliana*	*AtCDC48*	*cdc48* mutant impairs oleosin degradation, delaying post- germinative growth		Deruyffelaere et al., 2018
	CGI-58 (interacts with PXA1 to coregulate lipid homeostasis and signaling)	*A. thaliana*	*AtCGI58*	Interacts with PXA1 to coregulate lipid metabolism and signaling, particularly in nonseed vegetative tissues		Park et al., 2013

Plants possess multiple DGAT isoform, including the ER-localized DGAT1 and DGAT2, as well as a soluble DGAT3 whose physiological role remains under investigation (Qin et al., 2023). In *A. thaliana*, PDAT contains two homologs, and PDAT1 is the dominant isoform in TAG biosynthesis (Fan et al., 2019b). Distinct expression patterns and functional specializations among TAG-synthesizing enzymes enable plants to adjust lipid metabolism according to developmental and environmental cues. In different plant species, such as *A. thaliana*, *Camelina sativa* and soybean, *DGAT1* is the most highly expressed TAG biosynthetic enzyme (Hatanaka et al., 2022). Loss of AtDGAT1 activity in the *A. thaliana dgat1–1* mutant leads to a reduction in seed oil content by at least 20% (Katavic et al., 1995; Regmi et al., 2020), while the *dgat1–1 dgat2* double mutant does not display more oil reduction than *dgat1–1* mutant (Zhang et al., 2009). As for *PDAT* genes, either oil content or FA composition is affected by the *Atpdat1* mutation. The *AtDGAT1* mutation causes the up-regulated expression of *AtPDAT1*, and the *dgat1–1 pdat1–1* double mutant is lethal, indicating DGAT1 and PDAT1 have overlapping functions in *A. thaliana* TAG biosynthesis. The suppression of *AtPDAT1* expression by RNAi interference in the *dgat1–1* genetic background reduces oil accumulation by 70% to 80%, suggesting that PDAT1 rather than DGAT2 supports TAG biosynthesis when DGAT1 is lacking (Zhang et al., 2009). Furthermore, the detailed role of DGAT2, DGAT3 and PDAT2 in seed oil biosynthesis is unclear (Regmi et al., 2020).

In addition to TAGs, other forms of nonpolar lipids may also be present in LDs of some specific plant species. Wax esters (WEs), which are neutral lipids composed of a fatty alcohol esterified to a fatty acid. The WEs are synthesized through two enzymatic reactions catalyzed by fatty Acyl-CoA reductase and wax synthase. In jojoba (*Simmondsia chinensis*), a small shrub native to the deserts of North America, WEs can accumulate up to 60% of the seed weight (Sturtevant et al., 2020). Some algae, mosses, and pollen grains may also accumulate wax esters in LDs, though typically in smaller amounts (Guzha et al., 2023).

### 2.2 Lipid droplet proteins

Following their synthesis, neutral lipids — primarily TAGs — begin to accumulate between the leaflets of the ER membrane, forming small lens-like structures. These nascent lipid globules gradually expand through localized lipid synthesis and incorporation of additional neutral lipids. As these globules undergo expansion, they undergo a process of budding towards the cytosol, eventually maturing into discrete LDs (**Figure 1**, **Table 1**). This process of budding and stabilization is contingent on the recruitment of LD proteins. LD proteins are classified into two groups based on the pathways that they employ to traffic to LDs: class I LD proteins and class II LD proteins. Class I LD proteins are composed of proteins that are co-translationally inserted into the cytoplasmic face of the ER bilayer; in contrast, class II LD proteins target the LD from the cytoplasm (Mathiowetz and Olzmann, 2024).

#### 2.2.1 OLEOSIN, CALEOSIN and STEREOLESIN

The presence of OLEOSIN proteins on the phospholipid layer of LDs plays a crucial role in LD formation and its functional regulations (Anaokar et al., 2024). The prevailing LD proteins identified in the seeds of plants are OLEOSIN, CALEOSIN, and STEREOLESIN (Guzha et al., 2023). In *A. thaliana*, there are a total of 16 *OLEOSIN* genes, which include five seed-type *OLEOSIN* genes. Among these, *OLE1* is the most abundant *OLEOSIN* in *A. thaliana* seeds, followed by *OLE2*. OLEOSINs play a crucial role in preventing oil body fusion, thus maintaining the structural integrity of oil bodies. The OLEOSIN content is critical for oil body size regulation; a reduction in OLEOSIN content leads to an increase in oil body diameter due to the steric hindrance of OLEOSINs on the oil body surface inhibiting oil body fusion. Seeds of *OLEOSIN* single mutants (*ole1* and *ole2*) contain larger oil bodies than those of the wild type, and seeds of an *OLEOSIN* double mutant (*ole1 ole2*) contain even larger oil bodies than those of *ole1* and *ole2* single mutants. This suggests that OLEOSINs are essential for normal germination and enhance plant survival during winter by inhibiting freezing stress-induced oil body fusion (Siloto et al., 2006). Recent studies have identified a low-abundance, seed-specific LD protein termed LIPID DROPLET PROTEIN OF SEEDS (LDPS), which contains an amphipathic α-helix and a proline hairpin motif that serve as LD targeting signals. A distinct domain of LDPS mediates its interaction with OLE1. *ldps* mutant shows smaller LDs, reduction in seed oil content, and complete absence of LD fusion during post-germinative growth. Genetic analyses using *ole1* and *ldps* single mutants, double mutants, along with freeze-thaw experiments, demonstrated that OLE1 negatively regulates the LDPS-mediated promotion of LD expansion (Doner et al., 2025).

In comparison to OLEOSIN protein, CALEOSIN, which comprises three distinct domains, including N-terminal hydrophilic domains, C-terminal hydrophilic domains, and a central hydrophobic anchor domain, with the N-terminal domain containing a calcium-binding motif, exhibits a lower abundance (Shen et al., 2014; Liu et al., 2022). Moreover, from an evolutionary perspective, CALEOSIN protein exhibits homologous sequences in algae, fungi, and non-vascular plants, while such homology is not observed for OLEOSIN (Shen et al., 2014). The *A. thaliana* genome contains eight *CALEOSIN* genes divided into two groups: high-Mw *CALEOSIN* (*CLO1*, CLO2, *CLO3* and *CLO8*) and low-Mw *CALEOSIN* (*CLO4*-*LOL7*) (Liu et al., 2022; Miklaszewska et al., 2023). Several studies have indicated that CALEOSIN proteins have overlapping functions in oil accumulation (Shen et al., 2014; Liu et al., 2022; Miklaszewska et al., 2023). The STEREOLESIN-related proteins participate in intracellular signaling during plant growth and development by being involved in the phytohormone pathways, e.g. brassinosteroids-related pathways (Shao et al., 2019).

#### 2.2.2 Endoplasmic reticulum machinery: SEIPIN, LDIP, and VAP27

The SEIPIN complex, named after Berardinelli-Seip congenital lipodystrophy (BSCL), associates with these lipid lenses and directs the budding of nascent LDs into the cytoplasm. Most plants have multiple *SEIPIN* genes, in *A. thaliana*, three *SEIPIN* genes encode proteins with conserved structural features, predicted to form barrel-like complexes at the ER-LD junction (Arlt et al., 2022). Notably, AtSEIPIN2 and AtSEIPIN3 have longer N termini, with AtSEIPIN3 promoting the proliferation of very small LDs in leaves (Cai et al., 2015). The FFAT motifs present at the N termini of both SEIPIN2 and SEIPIN3 have been shown to interact with VAPs (Slee and Levine, 2019). VAPs, which are conserved across kingdoms, have been identified as the structural elements that facilitate contact sites between organelle membranes. The LD-forming complex has been demonstrated to be stabilized by VAP27–1 through a direct interaction with the N terminus of SEIPIN2 and/or SEIPIN3, a process that is deemed to be essential for LD biogenesis (Greer et al., 2020). In planta, loss of *VAP27–1* results in the formation of large LDs in seeds, a phenotype similar to that observed in *seipin2 seipin3* double mutants (Taurino et al., 2018). In addition, AtSEIPIN2 and AtSEIPIN3 are crucial for the modulation of the number and size of LDs by interacting with LDIP, facilitating LD biogenesis (Pyc et al., 2021).

## 3 Lipid droplet degradation through lipolysis by cytosolic lipases

In yeast, Drosophila, plants, and humans, stored TAGs are typically degraded by lipases, a conserved protein family with a patatin domain (Xu and Shanklin, 2016). As mentioned previously, lipolysis refers to the process of TAG degradation in LDs mediated by lipases, whereas lipophagy denotes an autophagic mechanism for LD degradation. The stored lipids are hydrolyzed by these lipases, leading to the breakdown of TAGs into components such as diacylglycerols (DAGs), monoacylglycerols (MAGs), fatty acids (FAs), and glycerol. Subsequent to this process, the hydrolytic byproducts enter diverse metabolic pathways, thereby playing pivotal roles in cellular growth, energy balance, and other physiological processes, occurring at the opportune moment (Kretzschmar et al., 2020). *SDP1*, the patatin-like acyl-hydrolase domain protein encoding gene, was discovered using forward genetic screening in *A. thaliana* (**Figure 1**) (Eastmond, 2006). Subsequent evidence suggests that this protein also serves as a primary enzyme for TAG hydrolysis in the leaves and roots of mature plants (Kelly et al., 2013a; Fan et al., 2014). During the early stages of seed germination in *A. thaliana*, SDP1 initially localizes to the surface of peroxisomes in an inactive form and subsequently extends to the surface of LDs within peroxisomes to hydrolyze TAGs (Thazar-Poulot et al., 2015). Further investigations have revealed that FYVE DOMAIN PROTEIN REQUIRED FOR ENDOSOMAL SORTING 1 (FREE1), within the ENDOSOMAL SORTING COMPLEX REQUIRED FOR TRANSPORT (ESCRT), directly interacts with both PEROXIN 11e (PEX11e) and SDP1, thereby facilitating the transport of SDP1 from peroxisomes to LDs (Huang et al., 2022). In addition to SDP1, *A. thaliana* possesses other patatin domain-containing lipases, such as SDP1-LIKE (SDP1L), which exhibit lipase activity and can release FAs from TAGs (Kelly et al., 2011, 2013a). Both SDP1 and SDP1L proteins are involved in the hydrolysis of TAGs during seed germination and also vegetative growth (Kelly et al., 2013a; Huang et al., 2022). In addition, the *AtOBL1* gene in *A. thaliana* encodes for an enzyme known as OIL BODY LIPASE 1, which is associated with LDs. AtOBL1 represents the only described TAG lipase from *A. thaliana* that is associated with LDs, as SDP1 is regarded as a peroxisome-associated protein (Müller and Ischebeck, 2018).

Besides, biochemical analysis indicates that SDP1 and SDP1L preferentially hydrolyze TAGs over DAGs and monoacylglycerols (MAGs). The purification of oil body membranes from *sdp1 sdp1L* double mutant seedlings revealed a deficiency in TAG lipase activity. However, the hydrolysis of DAGs and MAGs was still observed, indicating the presence of other lipid enzymes that function in synergy with patatin-like acyl-hydrolases to complete the hydrolysis of TAG (Eastmond, 2006; Kelly et al., 2011, 2013a).

Following the liberation of FAs into the cytoplasm by SDP1, these FAs are converted into CoA esters through the action of currently unidentified Acyl-CoA synthetases (Li et al., 2016). The subsequent translocation of FAs across the peroxisomal membrane is facilitated by PXA1, an ABCD transporter belonging to the ATP-binding cassette (ABC) transporter family. Notably, PXA1 exhibits a unique intrinsic Acyl-CoA thioesterase activity (De Marcos Lousa et al., 2013). This distinctive property enables PXA1 to first bind fatty Acyl-CoAs on the cytosolic face of the peroxisomal membrane, then cleave the CoA moiety, and ultimately mediate the import of free FAs into the peroxisomal matrix for *β*-oxidation - a metabolic process that yields Acetyl-CoA as the end product (Kunz et al., 2009; Li et al., 2016; Fan et al., 2017). In the case of impaired *β*-oxidation function, *pxa1* mutants exhibit delayed germination and reduced germination rate due to insufficient ATP supply required for the germination process. However, this defect can be alleviated by supplementing external carbon sources (Kunz et al., 2009). In addition, compared to the wild type, *pxa1* mutant shows increased sensitivity to dark conditions and exhibits early plant death due to the compromised *β*-oxidation (Fan et al., 2017).

In addition, COMPARATIVE GENE IDENTIFICATION-58 (CGI-58) protein positively regulates lipid metabolism through *β*-oxidation-related pathway. Chapman et al. identified the homologous gene of human *CGI58* in *A. thaliana*, referred to as *CGI58-like* (Yamaguchi and Osumi, 2009; James et al., 2010). In the *A. thaliana* mutant of this gene, plant leaves display a significantly increased TAG content of over tenfold compared to the wild type. However, unlike the *sdp1* mutants, germination and growth of *cgi-58* mutants do not show obvious defects (Yamaguchi and Osumi, 2009). Subsequent studies by Park et al. demonstrated that CGI-58 interacts with PXA1 to coregulate lipid homeostasis and signaling in *A. thaliana* (Park et al., 2013).

In the context of seedling establishment, the rapid breakdown of TAGs in planta, predominantly within four days, is particularly noteworthy. This phenomenon is further compounded by the accelerated degradation of LD-associated proteins, which may contribute to the enlargement of LDs during this critical phase. Studies have demonstrated that several LD proteins, including oleosins and steroleosins, have been observed to undergo polyubiquitination, a process associated with protein degradation (Deruyffelaere et al., 2018). This pathway, which is dependent on the removal of proteins from membranes, involves the action of the ubiquitin-proteasome system. Intriguingly, the analysis highlights the potential role of CELL DIVISION CYCLE PROTEIN 48 (CDC48) unfoldases, conserved in eukaryotes, in facilitating the unfolding and removal of membrane proteins. In planta, CDC48 has been observed to collaborate with PUX10, a scaffold protein residing at the LDs, to facilitate the degradation of ubiquitinated proteins (Kretzschmar et al., 2018; Liu et al., 2024). *pux10* mutants exhibit a reduced rate of LD protein degradation and an accumulation of ubiquitinated proteins (Deruyffelaere et al., 2018). However, to date, no known degradation mechanism has been identified for LD membrane lipids.

## 4 Autophagic degradation of lipid droplets

Lipophagy, a selective autophagic process, first described in mammals, is a process that involves the selective uptake of LDs into the vacuole or lysosome, followed by their degradation (**Figure 1**, **Table 1**). Notably, mammalian lipophagy is a form of macroautophagy, in which autophagosomes engulf LDs, distinguishing it from microlipophagy observed in yeast. Autophagy, a self-degradative and highly conserved process, plays a crucial role in various developmental processes within cellular organisms (Zhao et al., 2020). Autophagy primarily functions through vacuolar degradation and recycling of harmful or obsolete cellular components, thereby maintaining cellular homeostasis and facilitating adaptation to environmental changes (Avin-Wittenberg et al., 2012; Couso et al., 2018; Sun et al., 2018; Bao et al., 2020). The identification of *AUTOPHAGY-RELATED* (*ATG*) genes in *Saccharomyces cerevisiae* revolutionized our understanding of autophagy, revealing a highly conserved eukaryotic mechanism (Marshall and Vierstra, 2018). Subsequent studies identified homologous *ATG* genes in plants, including *A. thaliana, Oryza sativa*, and *Zea mays*, through sequence alignment analyses (Li et al., 2015). These studies uncovered over 40 evolutionarily conserved ATG proteins that orchestrate autophagosome biogenesis and autophagy regulation across kingdoms, from yeast to mammals and plants (Marshall and Vierstra, 2018).

In *A. thaliana*, two independent studies, Fan et al. and Havé et al., reached the same conclusion through different approaches, thereby demonstrating the involvement of autophagy in the degradation of lipids (Fan et al., 2019a; Havé et al., 2019). Their findings suggest that, in *A. thaliana* leaves, basal autophagy contributes to TAG synthesis, whereas inducible autophagy under starvation contributes to LD degradation (Fan et al., 2019a). Besides, direct evidence through ultrastructural analysis has demonstrated that LDs are degraded in autophagic vacuoles (Fan et al., 2019a). In the parallel study, Havé et al. utilized protein and lipid profiling analyses on *atg5* mutant, demonstrating that autophagy plays a pivotal role in the lipid metabolism of the ER and peroxisome in *A. thaliana* leaves (Fan et al., 2019a; Havé et al., 2019). Fan et al. investigated the role of autophagy in lipid metabolism by using mutants with auto (Havé et al., 2019). In addition to its role in *A. thaliana*, autophagy has been observed to contribute to the degradation of LD in other plant species. In rice, investigating *osatg7* mutants has demonstrated autophagy’s crucial role during the late stages of pollen meiosis. As LDs are critical for energy supply, *osatg7* mutants exhibit reduced levels of autophagy, and such deficiency leads to impaired pollen maturation (Kurusu et al., 2014).

## 5 Lipid droplets are involved in abiotic and biotic stress responses

### 5.1 Abiotic stress

It is imperative to acknowledge that plants are subject to numerous stressors throughout their life cycle, which necessitates the orchestration of adaptive responses to these environmental cues by all cellular organelles. Among these organelles, cytosolic LDs and their core set of neutral lipids and associated surface proteins play a significant yet understudied role. It has been demonstrated that environmental changes have a substantial influence on LD-related processes. For example, the abundance of LDs in *A. thaliana* leaves increases under drought, cold, or heat stress (Yang et al., 2011; Kong et al., 2013; Kim et al., 2016; Yang et al., 2024).

A close relationship exists between stress and TAG accumulation in plant tissues, especially the vegetative tissues (Lee et al., 2019). For instance, low-nitrogen stress and the stress hormone abscisic acid (ABA) have been observed to stimulate TAG accumulation in *A. thaliana* seedlings (Yang et al., 2011; Kong et al., 2013; Coulon et al., 2024). During periods of heat stress, cells undergo a process of unsaturated acyl chain replacement with saturated ones, a process that may lead to an increase in membrane fluidity (Mueller et al., 2017; Yang et al., 2024). This phenomenon suggests that LDs may absorb discarded unsaturated acyl chains from membrane lipids, resulting in the formation of triacylglycerols, thereby facilitating membrane remodeling (Yang et al., 2011; Scholz et al., 2025). Transgenic plants overexpressing *LIPID DROPLET-ASSOCIATED PROTEINS* (*LDAPs*) exhibit enhanced drought tolerance, suggesting a close relationship between stress and TAG accumulation in vegetative tissues (Zhao et al., 2023).

It has been determined that ABA signaling plays a pivotal role in the regulation of LD generation, particularly with regard to the expression of *DGAT1*. Tobacco transient assays have revealed a synergistic effect of ABA-insensitive 4 (ABI4) and ABI5, two important ABA-related transcription factors, in regulating *DGAT1* expression under stress (Yang et al., 2011; Kong et al., 2013). Furthermore, a comprehensive transcriptome analysis has revealed that *LIPID DROPLET PROTEIN* (*LDP*) genes, including *OLEOSINs* and *CALEOSINs*, exhibited up-regulation of up to 1000-fold through the activation of ABI3. This provides compelling genetic evidence that ABI3 activates oil accumulation, most likely through up-regulating *LDPs* (Yang et al., 2022).

### 5.2 Biotic stress

Furthermore, LDs have been observed as targets by invasive organisms. *Phytophthora infestans* degrade LDs as energy source in guard cells to maintain stomatal opening (Yang et al., 2021). Plant RNA viruses induce endomembrane proliferation for viral replication compartments (VRCs) formation, and the host lipid metabolism is crucial for their replication. However, to date, direct links between LDs and plant virus infection have not been firmly established, and the extent of their involvement in plant defense or viral benefit remains to be elucidated (Zhang et al., 2019). In addition, during infections by pathogens such as *Botrytis cinerea* or *Pseudomonas syringae*, the leaves of plants exhibit an increased accumulation of TAGs (Sham et al., 2014; Galluzzi and Green, 2019). Besides, LDs have been proposed to function as “subcellular factories” for the production of antimicrobial compounds. For instance, two key lipid-modifying enzymes - peroxygenase (CLO3) and α-DIOXYGENASE (α-DOX) - coordinately catalyze a coupling reaction that converts α-linolenic acid into the antifungal compound 2-hydroxy-octadecanoic acid during defense responses against *Colletotrichum higginsianum* infection (Fernández-Santos et al., 2020). *LDAP1*, *CLO3*, and α*-DOX1* are upregulated in leaves infected by *Botrytis cinerea*, suggesting that LD biosynthesis is induced either by the fungi or as a plant defense mechanism. The fatty acid composition of TAGs varies depending on the infecting pathogen, indicating the presence of distinct synthesis pathways. The hijacking of LDs by pathogens or their utilization by plants for defense mechanisms bears resemblance to the processes observed in animal cells (Roingeard and Melo, 2017). Meanwhile, PHYTOALEXIN DEFICIENT 3 (PAD3), a cytochrome P450 monooxygenase known to be involved in the biosynthesis of antimicrobial phytoalexins, has been observed to translocate to LDs following infection by *Pseudomonas syringae* (Fernández-Santos et al., 2020). This dynamic relocation of defense-related enzymes to LDs highlights the organelle’s emerging role as a critical platform for organizing plant immune responses.

## 6 Conclusions and prospects

This review offers a detailed examination of the processes involved in the formation and breakdown of LDs in plants, emphasizing crucial enzymes, regulatory pathways, and physiological contexts. A more profound understanding of the regulatory mechanisms governing LD-associated pathways holds considerable potential for enhancing crop yield and promoting bioenergy production. Manipulating the genes and proteins involved in LD biogenesis and turnover may lead to the development of crops with higher oil yields and improved stress resilience.

Many studies have demonstrated that LDs play a crucial role in cellular lipid homeostasis. While much attention has been paid to how LD size and number are determined in plants (Doner et al., 2025), the regulation of lipolysis and lipophagy-mediated lipid turnover, particularly during nutrient deprivation when plants rely on lipid catabolism for energy production, remains poorly understood. In mammals, the process of lipolysis is subject to stringent regulation, with the rate-limiting enzyme Adipose Triglyceride Lipase (ATGL) being subject to enhancement of up to 20-fold through its interaction with the activator CGI-58 (ABHD5) (Mathiowetz and Olzmann, 2024). Concurrently, PLIN proteins function as a regulatory mechanism, sequestering CGI-58 and thereby impeding ATGL activity (Mathiowetz and Olzmann, 2024). In contrast, plants employ SDP1 as their functional ATGL homolog, but lack both PLIN proteins and CGI-58-mediated activation of SDP1, despite the presence of a CGI-58 homolog that instead regulates PXA1 (Park et al., 2013). The current understanding of plant lipolysis regulation remains incomplete, particularly regarding whether energy-sensing pathways modulate SDP1 activity. The energy-sensing central regulators include the low-energy sensor SnRK1, the high-energy sensor TOR kinase and the sucrose-signaling metabolite T6P. These components form an intricate regulatory network where SnRK1 promotes lipolysis during energy deficit while TOR suppresses it under energy-replete conditions, with T6P fine-tuning this balance by inhibiting SnRK1 (Figueroa and Lunn, 2016; Liu and Xiong, 2022; Van Leene et al., 2022). Critical areas for future investigation include investigating the possible direct phosphorylation of SDP1 by SnRK1/TOR kinases, characterizing the functional relationship between the energy sensing module and SDP1 during lipid mobilization, and identifying potential novel components that facilitate communication between SDP1 and energy-sensing pathways. Resolution of these questions will significantly advance our understanding of the molecular mechanisms controlling LD degradation and overall plant lipid homeostasis.

Current research indicates that plants may dynamically regulate the functions of LD-associated proteins through post-translational modifications (PTMs) in response to environmental stresses. Despite extensive characterization of LD protein PTMs in animal systems (Zhang et al., 2022; Loix et al., 2024), their functional validation and molecular mechanisms remain largely unexplored in plants. Ubiquitination may regulate LD protein turnover through either proteasomal degradation or selective autophagy (e.g., lipophagy), maintaining cellular homeostasis under stress conditions. Furthermore, oxidative modifications and SUMOylation likely participate in mediating LD-organelle interactions (e.g., with peroxisomes), affecting membrane remodeling and ROS scavenging. Future investigations should integrate subcellular proteomics, PTM site-directed mutagenesis, and super-resolution imaging to systematically decipher stress-specific PTM dynamics on LD proteins and their physiological relevance. Such advances would not only elucidate the regulatory mechanisms of plant lipid metabolism under stress but may also provide novel strategies for improving crop stress tolerance.

LDs serve as critical organelles in stress response mechanisms. Numerous abiotic stressors have been shown to induce LD biogenesis (Zhao et al., 2023; Coulon et al., 2024). During senescence or stress conditions, TAG accumulation is closely linked to lipid catabolic processes. However, several key aspects remain poorly understood: the functional significance of fatty acids derived from membrane lipids like Monogalactosyldiacylglycerol (MGDG) (Fan et al., 2017); the specific roles of various lipases in stress adaptation; and the degradation mechanisms of stress-induced LDs during post-stress recovery. While LD degradation during seed germination has been well characterized (Kelly et al., 2011), the catabolic pathways of stress-induced LDs and their contributions to cellular homeostasis restoration remain elusive. Particularly, the relative importance of lipolysis versus lipophagy in TAG remobilization, the metabolic fates of neutral lipids, and the subsequent utilization of released fatty acids all require systematic investigation (Coulon et al., 2024). Elucidating these processes will not only advance our understanding of plant stress responses but also provide a theoretical framework for developing stress-resistant crops through LD manipulation.

Research has shown that the proteome of LDs in plants undergoes extensive dynamic remodeling under diverse stress conditions (Krawczyk et al., 2022). This is evidenced by the specific upregulation of stress-responsive LD-associated proteins, such as CLO3 and α-DOX1, in both wild-type plants and the *tgd1–1 sdp1–4* mutant (Shimada et al., 2014). Notably, different stresses exhibit distinct regulatory effects on LD proteins: CLO3 responds to both heat stress and pathogen infection, whereas α-DOX1 is selectively activated only under pathogen infection and drought stress conditions (Scholz et al., 2025). These findings suggest that plants have evolved a stress-specific LD reprogramming mechanism, fine-tuning protein expression to adapt to different environmental threats. However, the molecular mechanisms governing LD remodeling under various stress conditions remain poorly understood and warrant further investigation.
