# Correction: Lipid droplets in plants: turnover and stress responses

**DOI:** 10.3389/fpls.2025.1727402

**Published:** 2025-10-27

**Authors:** Yujie Zhao, Rui Cao, Jincheng Li, Yingying Xu, Lijuan Zhou, Yajin Ye

**Affiliations:** State Key Laboratory of Tree Genetics and Breeding, National Key Laboratory for the Development and Utilization of Forest Food Resources, Co-Innovation Center for Sustainable Forestry in Southern China, Nanjing Forestry University, Nanjing, China

**Keywords:** lipid droplets, biogenesis, degradation, stress responses, SDP1, lipophagy

In the published article, there was a mistake in the **Funding** statement. The **Funding** statement for Natural Science Foundation of Jiangsu Province was displayed as “BK2022045268 to LJ Zhou, BK20220417 to YJ Ye”. The correct statement is “Natural Science Foundation of Jiangsu Province (BK20220418 to LJ Zhou, BK20220417 to YJ Ye).’’

The original version of this article has been updated.

